# Supplementary material for: ACE2 gene variants may underlie interindividual variability and susceptibility to COVID-19 in the Italian population
Source: Eur J Hum Genet. 2020 Jul 17;28(11):1602–14. doi: 10.1038/s41431-020-0691-z (PMC7366459; doi:10.1038/s41431-020-0691-z)
Supplement: Supplementary file 1 — Supplemental Material [file 41431_2020_691_MOESM1_ESM.docx]

**Supplemental Material**

**Supplementary Table S1. *ACE2* intronic and exonic variants**

The supplementary table reports *ACE2* intronic and exonic variants identified in 6930 Italian exomes. The genomic reference sequence is NM_021804.2 (hg19). The variant recurrency in unrelated individuals is reported. Heterozygous individuals are indicated with 0/1 count while homozygous individuals are indicated with 1/1 count. When available, dbSNP rs number and the genome aggregation database gnomAD allele frequency are reported.

**Supplementary Figure S1. Conservation of amino acid residues among species**

The supplementary figure reports the conservation among species of three common variants ((p.(Gly211Arg), p.(Asn720Asp) and p.(Lys26Arg)) and three rare variants (p.(Leu351Val), p.(Pro389His) and p.(Val506Ala)) using Alamut Software V2.11.

**Supplementary video S1. *ACE2* Val506Ala simulation**

**Supplementary video S2. *ACE2* Gly211Arg simulation**

**Supplementary video S3. *ACE2* Lys26Arg simulation**

**Supplementary video S4 *ACE2* Leu351Val simulation**

**Supplementary video S5 ACE2 Pro389His simulation**
