# Supplementary material for: ACE2 gene variants may underlie interindividual variability and susceptibility to COVID-19 in the Italian population
Source: Eur J Hum Genet. 2020 Jul 17;28(11):1602–14. doi: 10.1038/s41431-020-0691-z (PMC7366459; doi:10.1038/s41431-020-0691-z)

g.1517T>C p.Val506Ala

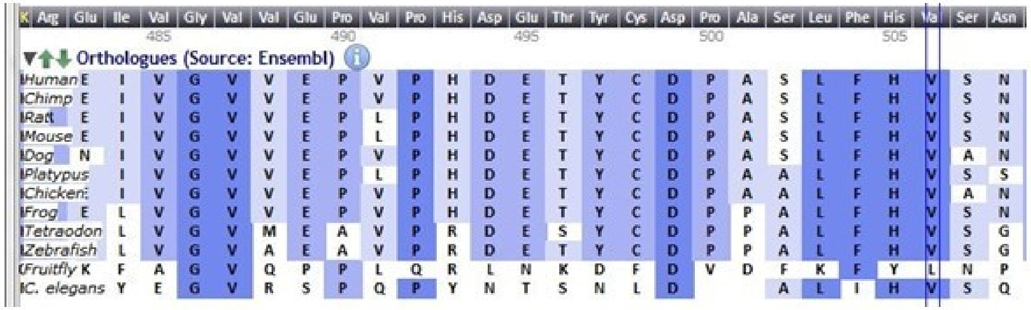

c.1051C>G p.Leu351Val

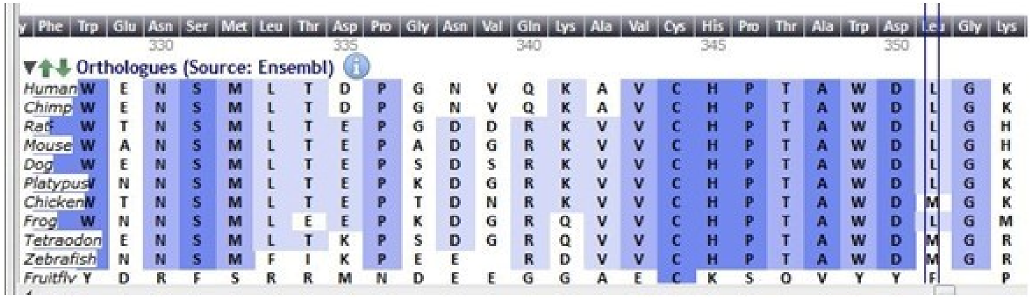

c.77A>G p.Lys26Arg

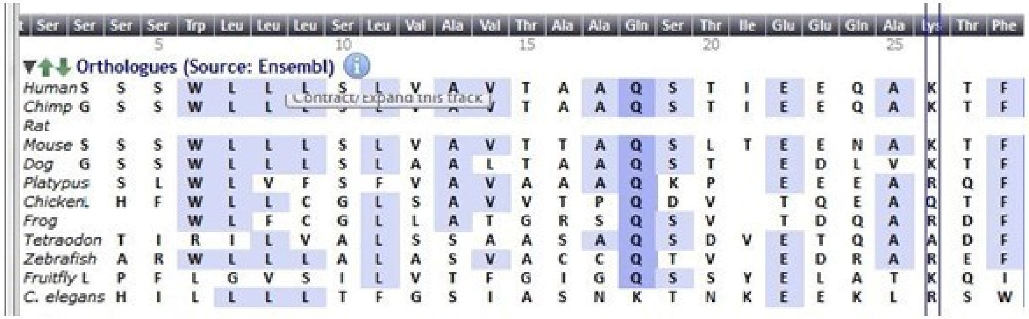

c.1166C>A p.Pro389His

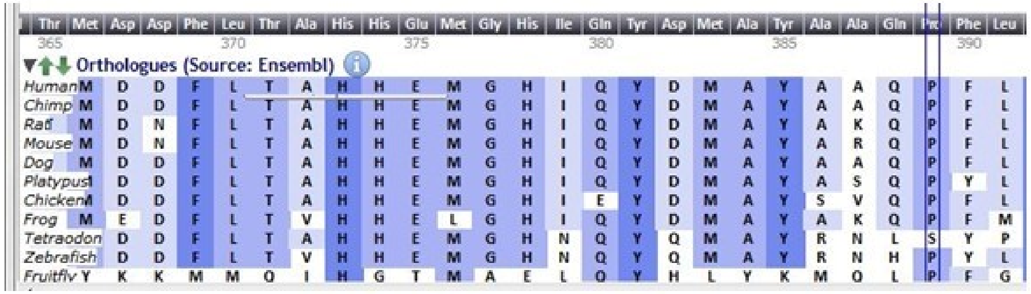

c.631G>A p.Gly211Arg

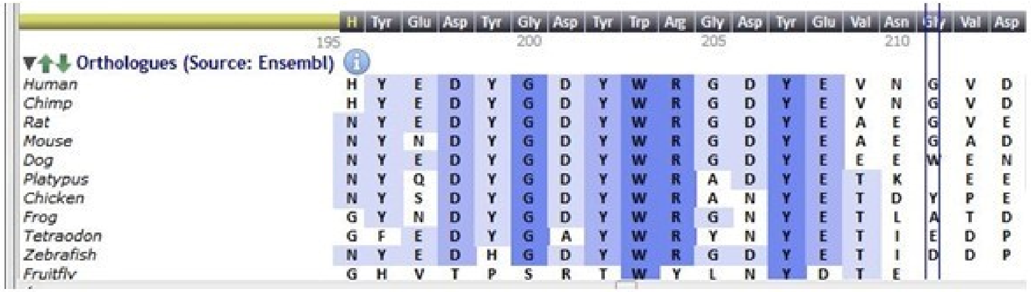

c.2158A>G p.Asn720Asp

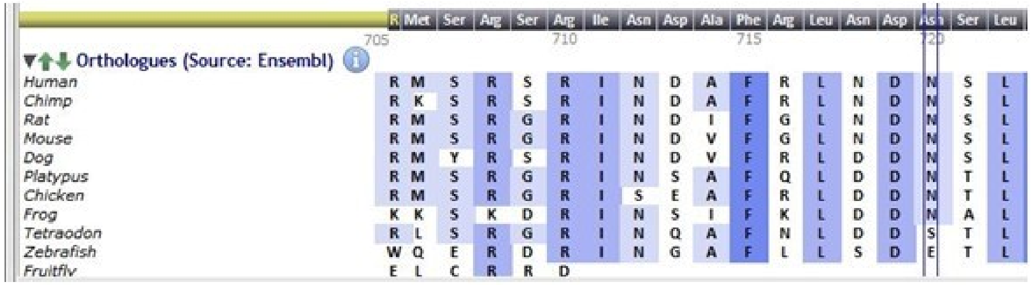

Supplement: Supplementary file 7 — Supplementary Figure S1 [file 41431_2020_691_MOESM7_ESM.pdf]
